# Leiomyosarcoma of the inferior vena cava: Radical surgery and vascular reconstruction

**DOI:** 10.1186/1477-7819-7-56

**Published:** 2009-06-26

**Authors:** Andrea Alexander, Alexander Rehders, Andreas Raffel, Christopher Poremba, Wolfram T Knoefel, Claus F Eisenberger

**Affiliations:** 1 Klinik für Allgemein-, Viszeral- und Kinderchirurgie, Universitätsklinikum Düsseldorf, Germany; 2 Institut für Pathologie, Universitätsklinikum Düsseldorf, Germany

## Abstract

**Background:**

Vascular leiomyosarcoma are rare tumors typically originating from the inferior vena cava (IVC). Due to nonspecific clinical signs most tumors are diagnosed at advanced stages. Complete surgical resection remains the only potential curative therapeutic option. Surgical strategy is particularly influenced by the level of the IVC affected. Due to the topographic relation to the renal veins level-II involvement of the IVC raises special surgical challenges with respect to the maintenance of venous outflow.

**Case presentation:**

We herein report two cases of leiomyosarcoma of the IVC with successful en bloc resection and individualized caval reconstruction. One patient presented with a large intramural and intraluminal mass and received a complete circumferential resection. Reconstruction was performed by graft replacement of the caval segment affected. The other patient displayed a predominantly extraluminal tumor growth and underwent semicircumferential resection of the IVC including the confluence of the left renal vein. In this case vascular reconstruction was performed by cavoplasty and reinsertion of the left renal vein into the proximal portion of the IVC. Resection margins of both patients were tumor free and no clinical signs of venous insufficiency of the lower extremity occurred.

**Conclusion:**

This paper presents two cases of successfully managed leiomyosarcomas of the vena cava and exemplifies two different options for vascular reconstruction in level II sarcomas and includes a thorough review of the literature.

## Background

Primary vascular leiomyosarcoma is a rare tumor with less than 300 cases reported. It originates from the smooth muscle cells of the media and predominantly arises within the inferior V. cava (IVC) [1]. While intraluminal tumor growth is rarely found, most patients present with extraluminal tumor growth along the adventitia of the IVC [2]. The origin of the tumor is described in relation to the hepatic and renal veins. For this purpose, the IVC is divided into three levels: level 1 extends from the entry of the hepatic veins up to the right atrium, level 2 comprises the area between the confluences of the renal and hepatic veins whereas level 3 includes the area below the renal veins. Actually, level 2 of the IVC is most frequently affected [3-5].

Due to the absence of early symptoms, retroperitoneal tumors are often not diagnosed until the disease is at an advanced stage with large tumor growth and involvement of surrounding structures. Clinical symptoms are unspecific, and most patients present with abdominal or flank pain [3], which is potentially accompanied by lower extremity edema due to deep vein thrombosis. Further symptoms include testicular swelling and shortness of breath [5]. Imaging modes such as color Doppler ultrasonography, contrast enhanced computed tomography or magnetic resonance imaging significantly contribute to the diagnosis.

By reason of the poor long-term prognosis and the surgical risk, the involvement of large vessels has traditionally been considered a limiting factor for resection of retroperitoneal tumors [6]. Yet advances in both surgical techniques and perioperative care have made major vascular surgery a safe therapeutic option for these patients [7]. Currently, radical en bloc resection of the affected venous segment remains the only therapeutic option associated with prolonged survival [3,8]. In a recent study on 20 patients with leiomyosarcoma of the IVC, radical surgery combined with adjuvant multimodal therapy yielded a 5-year cumulative survival rate of 62% [5].

However, the surgery that is required to accomplish complete tumor resection is challenging. The goals of surgical management of these tumors include the achievement of local tumor control, maintenance of caval flow, and the prevention of recurrence. The surgical strategy, however, is not only influenced by the level of the caval segment that is affected, but also by the extent of retroperitoneal collateral circulation, and by the topographic involvement of neighboring structures. In particular, the involvement of renal or hepatic veins dictates the strategy for vascular reconstruction.

The surgical management of partial resections of the IVC is a matter of current debate and includes ligation, primary repair/cavoplasty, or replacement with a graft. Reconstruction of the IVC is not always required, because gradual occlusion of the IVC allows the development of venous collaterals. However, when pararenal leiomyosarcoma of the IVC is present, reconstruction of the IVC and the renal vein is necessary to prevent transient or permanent renal dysfunction [9].

We herein report two cases of leiomyosarcoma of the IVC with emphasis on the surgical procedure and reconstruction of caval continuity.

## Case presentation

### Patient 1

**Figure 1:**
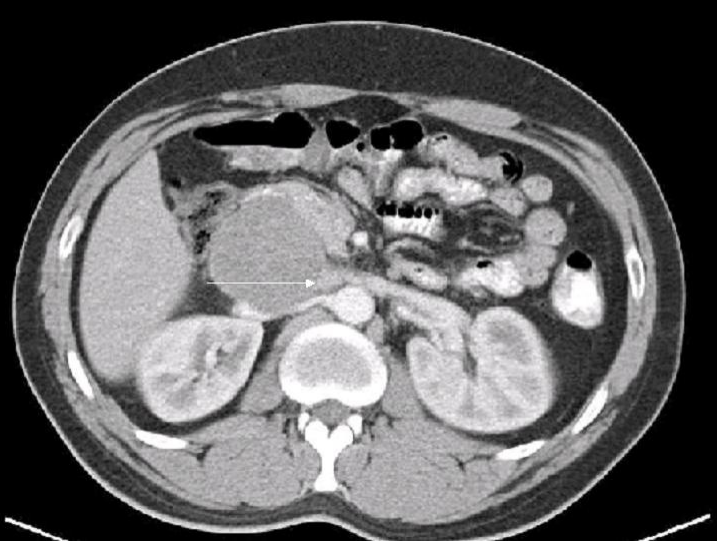
**Abdominal CT-scan**. Axial contrast enhanced CT image showing a retroperitoneal tumor with obstruction of the IVC and involvement of the left renal vein (arrow).

For more than seven years a 34-year old male patient had been complaining about recurrent discomfort of the upper abdomen and pain emanating to his back. Due to an increase of the preexisting disorders an abdominal CT-scan was conducted, which showed a cystic retroperitoneal tumor (figure 1).

**Figure 2:**
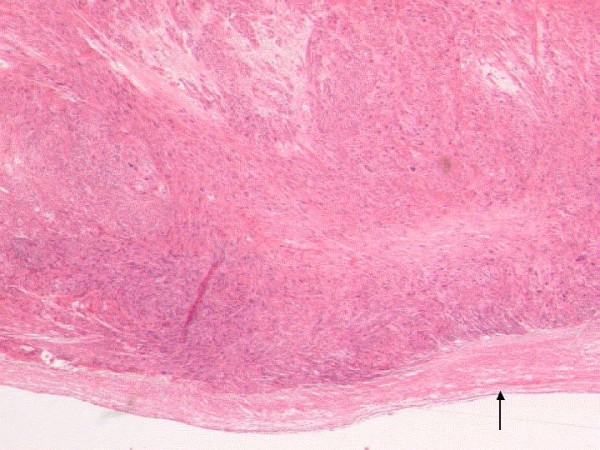
**Histopathologic specimen (HE stain, ×20)**. Spindle-cell tumor infiltrating the blood vessel wall with primarily extraluminal growth. In the lower part of the picture, part of the intimal layer of the blood vessel can be recognized (arrow).

The patient was referred to us with the suspicion of a pancreatic neoplasia. After reviewing the CT scans we suspected a retroperitoneal neoplasia and completed staging which showed no distant metastases. An exploratory laparotomy was performed. However, no pancreatic mass was palpable intraoperatively. After mobilization of the right hepatic lobe and the right colic flexure, the tumor was located between the inferior vena cava and the left renal vein compressing and infiltrating those vessels. To obtain cranial and caudal control, the IVC was longitudinally exposed and secured with vessel loops. Subsequently, the IVC was gradually clamped. Prior to exclusion of the venous segment the patient received heparin intravenously. Due to the predominantly extravascular tumor growth (figure 2) partial semi circumferential resection of the inferior vena cava, including the confluence and approximately 1.5 centimeters of the terminal left renal vein was performed and the tumor was resected 'en bloc'.

Intraoperative assessment of the surgical margins by frozen section confirmed that a complete R-0 resection had been achieved. Vascular reconstruction was performed by a running suture of the caval resection margin and by reinsertion of the left renal vein into the proximal portion of the IVC at the site of the suture, since the length of the remaining left renal vein was sufficient after mobilization for a tension-free anastomosis. Intraoperative duplex ultrasound of the cavoplasty presented a good venous flow in the reconstruated vessels.

The postoperative course was uneventful, without impairment of the renal function or swelling of the lower limbs. Due to the vascular surgical procedure, the patient was therapeutically anticoagulated with heparin. Histopathology revealed a lowly differentiated leiomyosarcoma with an extension of approximately 7 cm in diameter; the resection margins were tumor-free. After interdisciplinary discussion of the case adjuvant radiotherapy was conducted with 57.4 Gy in particular due to the advanced tumor size.

During the first follow-up after three months, the patient presented in good general condition. A CT-scan of the thorax and an abdominal MRI showed no indication for tumor recurrence. Under sustained anticoagulation with warfarin, the reconstructed vessels constituted without any pathological findings. Yet after six months the patient was still free of local recurrence, however, CT-scan showed a central hepatic lesion. A subsequently performed explorative laparotomy revealed inoperable disseminated hepatic metastases. According to interdisciplinary consent, the patient received regional hyperthermia as well as chemotherapy including ifosfamide, adriamycine, and etoposid during the following months.

### Patient 2

We furthermore report on the case of a 58-year-old female patient who was referred to our department with a retroperitoneal tumor which was diagnosed in an external hospital. This finding had resulted from an abdominal ultrasound, which was carried out because of unspecific abdominal pain. A CT-scan described a tumor that likely originated from the IVC, however not being clearly distinguishable from the right adrenal gland.

After mobilization of the liver, including segment one, the subsequent intraoperative finding revealed a large inferior caval tumor with an infrahepatic suprarenal localization that was consistent with the radiological statement. The tumor could not reliably be separated from the right adrenal gland. Thus, apart from a partial resection of the IVC, a right *adrenalecomy en bloc *was required. In contrast to patient 1, this tumor presented with a larger proportion of intravascular growth. Therefore, adequate oncologic resection required a complete circumferential resection of the IVC in addition to a right adrenalectomy *en bloc*, which was performed. Because the caval confluences of the renal veins were unaffected by the tumor on both sides, approximately 3 cm of the entire circumference of the infrahepatic IVC were resected as far as slightly above the renal confluences. Due to the low pressure in the IVC, the vascular continuity was reconstructed using a ring-enforced PTFE prosthesis 19 mm in diameter to optimize caval flow.

Because of the exogenous material implanted, the patient also received effective anticoagulation with heparin. On the second postoperative day, a relaparotomy was required under clinical suspicion of a secondary hemorrhage. Whereas this was confirmed, and hematomas in the right upper abdomen as well as within the omental bursa were detected, exploration of the entire abdomen could not reveal an origin of the bleeding. In particular, the exposure of the prothesis, including the anastomosis showed proper conditions. Thus, besides the removal of hematoma with abdominal lavage, no further intervention was performed. During the following course the patient recovered without complications.

Histopathological examination showed a moderately differentiated caval leiomyosarcoma. with a maximum extension of 4 cm in diameter, and almost complete luminal obliteration, as well as tumor-free resection margins. In compliance with interdisciplinary consent, no adjuvant therapy was indicated. The follow-up examinations currently lasting up to a year after surgery showed no signs of tumor recurrence.

## Discussion

Curative surgical resection remains the current treatment of choice for primary leiomyosarcoma of the IVC. A major surgical issue is the need for venous reconstruction. Basically, ligation of the IVC, cavoplasty, and graft replacement represent the major therapeutic options. However, surgical strategy is particularly influenced by the level of the IVC affected. When dealing with the infrarenal level 3 of the IVC, simple ligation has been found to yield good functional results with the assumption that the slowly growing tumor allows sufficient collaterals to develop. After extensive curative resection with disruption of collaterals or if only few collaterals have developed before surgery, however, ligation of the IVC may cause lower limb edema with significant functional impairment [10]. We consequently cannot propose caval ligation for patients with tumors of the cava, in particular of level II. Tumors that involve level II raise special challenges for operative treatment with respect to vascular reconstruction. Actually, the topographic relation to the renal veins is critical for surgical strategy maintaining venous outflow. The cases reported examplify two different options for vascular reconstruction in level II sarcomas. Patient 1 presented with predominantly extraluminal tumor growth, whereas patient 2 had a suprarenal leiomyosarcoma with a large intramural and intraluminal tumor mass. Due to the high-grade caval obliteration and the suprarenal location in this case, a complete circumferential resection with subsequent graft replacement was performed. Because the tumor in case one displayed a mainly extravascular growth that spread along the caval adventitia, a cavoplasty was performed instead of a circumferential resection. Furthermore, this strategy sustained the physiologic caval continuity and facilitated the reinsertion of the left renal vein.

Actually, there is considerable controversy about the type of caval reconstruction. Several authors recommend prosthetic replacement, but others often perform cavoplasty or ligation of the IVC [5]. In level II tumors, ligation of the IVC is precarious because impairment of the renal venous outflow might occur, resulting in renal dysfunction. This is particularly true for the right kidney. In case the right renal vein needs to be sacrificed, which is rarely the case, the kidney may loose its function and potentially has to be resected. In contrast, the left renal vein provides more collaterals after ligation close to the cava. But even on the left side renal functional impairment was described after sacrifice of the left vein [9]. For this reason we always reconstruct both renal veins after resection if at all possible.

If reconstruction of the renal vessels is possible and complete resection of the tumor is achieved, the surgeon should try to maintain renal function.

Prosthetic replacement, which enables circumferential resections is favored because of a more radical approach. This technique is predominantly applied in patients with large intramural and intraluminal tumors. However, this procedure, at least theoretically, carries an increased risk of pulmonary embolism as well as further graft-related major complications such as sepsis, graft occlusion and graft-enteric fistulas [4,7]. Particularly if radiation therapy is to be administered, entero-prosthetic fistulas are even more likely to develop [11].

So far there is neither evidence for an increased risk of thromboembolic complications after prosthetic replacement nor for a protective impact by effective anticoagulation. Whether long term anticoagulation is truly required is still under debate since it also carries the potential of hemorrhage. Yet cavoplasty and in particular foreign material in low flow venous segments predispose to thrombosis and potential embolization. Several cases of postoperative graft occlusions were reported [7,12]. Thus, we routinely perform anticoagulation for the low risk of complications in our experience.

The use of arterio-venous-fistulas represents another possibility to optimize caval patency. However, the effect of this procedure is yet controversial, since the potential benefits might not outweigh the risks. Complications such as cardiac insufficiency or local complications should be considered. Besides, we share the view of several authors stating that arterio-venous-fistulas are not required if the suprarenal IVC is reconstructed because of the high-volume blood flow at this level [9,12].

Leiomyosarcoma is reported to have a poor prognosis. Over half of patients who underwent radical resection develop tumor recurrence, and the 5-year survival rate ranges between 31 and 62% [5].

To improve the outcome of patients with leiomyosarcoma of the IVC, adjuvant and neoadjuvant treatment protocols are applied with promising results [8]. However, randomized trials concerning these options do not exist due to the heterogeneity of the cohorts and the rarity of the disease, the role of adjuvant or neoadjuvant therapy is yet uncertain. Depending on present risk factors such as age, the status of the resection margins, grading and particularly the tumor size [5,13] adjuvant radiotherapy is administered at our institution. In case 1 with a mainly extraluminal tumor growth and a large tumor size of 7 centimeters adjuvant radiotherapy was applied, whereas patient 2 with an intraluminal tumor measuring 4 centimeters limited to the vessel received no adjuvant treatment. Interestingly, the risk of local or distant recurrence is not found to be influenced by the extent of IVC resection as long as a complete resection can be achieved [1,14]. The type of IVC resection should be tailored individually depending on the topographic tumor expansion. Therefore, circumferential IVC resection requiring graft replacement is not obligatory. This concept was reflected in the treatment of the two patients reported on.

## Conclusion

Surgery, whether performed alone or in combination with adjuvant therapy constitutes the only hope of prolonged survival. For this reason, we recommend aggressive surgical management by using modern vascular surgical and oncological techniques.

## Consent

Written consent was obtained from the patients for publication of these case reports. A copy of the consent is available with Editor in Chief.

## Competing interests

The authors declare that they have no competing interests.

## Authors' contributions

AA was involved in the conception, design and preparation of manuscript, literature review, substantial intellectual contribution. ARe was involved in manuscript preparation and thorough review of manuscript, literature review and substantial intellectual contribution. Ara was involved in the initiation of report, critical revision of manuscript. CP was involved in the histopathological work up and contribution to pathology part of manuscript. WTK was involved in the initiation of report, performed surgery, through review and drafting of manuscript, substantial intellectual contribution. CFE was involved in the initiation of the report, important intellectual contribution drafting and review of manuscript. All authors read and approved the final manuscript.
